# Increasing creative self‐efficacy: Developing the confidence of biochemistry undergraduates to innovate

**DOI:** 10.1002/bmb.21628

**Published:** 2022-04-23

**Authors:** Simon Mark Payne, David Edward Whitworth

**Affiliations:** ^1^ Department of Psychology, Faculty of Earth and Life Sciences Aberystwyth University Aberystwyth Ceredigion UK; ^2^ Institute of Biological, Environmental, and Rural Sciences, Faculty of Earth and Life Sciences Aberystwyth University Aberystwyth Ceredigion UK

**Keywords:** creativity, employability, innovation, social scaffolding

## Abstract

Biochemistry graduates need to be creative, however assessing creativity requires the production of novelty, judged by or against that of peers. A related phenomenon is ‘creative self‐efficacy’ (CSE) – one's self‐belief in producing creative outcomes. CSE is a contributor to creativity, but is more easily assessed, and thus more amenable for targeting pedagogically. To investigate interactions between student CSE and the learning environment, a biochemistry laboratory exercise was deployed within a ‘creative’ module, wherein students created their own experimental protocols. Students completed questionnaires at the beginning and end of the module. Compared to ‘control’ modules lacking overtly creative activities, the creative module significantly increased students' perceptions of their own creativity and whether their studies had increased their creativity. Students' confidence in meeting degree learning outcomes (for instance the ability to work productively in a laboratory), and motivation to study, were also significantly increased. Marks attained from the creative exercise correlated with students' CSE, but surprisingly, students' expected marks correlated negatively with their CSE, implying they had a poor understanding of the relationship between creativity and success. Our results suggest that the learning environment can positively affect students' CSE, promoting academic attainment of learning outcomes, motivation, and their confidence as biochemists.

## 1 INTRODUCTION

Biochemistry educators want their graduates to be curious, critical appraisers of information, able to produce balanced rationales for investigations. These attributes are enhanced by creativity, which is defined as the ability to create novel, appropriate, and useful/impactful ideas and/or products.^1^, ^2^ Employers place a premium on innovation and creativity, which is reflected by their inclusion in employer‐informed degree accreditation criteria such as those offered by the Royal Society of Biology.^3^ Nevertheless, arts students typically score higher than STEM students on self‐rated creativity,^4^ therefore, STEM students can benefit from an improved appreciation of their own creativity, increasing their competitiveness for employment.

### 1.1 Creative self‐efficacy (CSE)

Creative self‐efficacy (CSE) is defined as ‘one's perceived ability to create novel and useful ideas and/or products’,^5^ and is positively associated with achievement in higher education.^6^ CSE differs fundamentally from creativity in that it is the *perception* of one's creativity, and is thus dependant on a student's beliefs about their ability to achieve mastery of their academic activities. Demonstrating creativity requires the production of novel ideas/products, which is challenging to assess objectively, as it must be judged by peers, or against that of peers.^7^ However, CSE is comparatively easy to quantify, for instance using a questionnaire for students to self‐report their perceived creativity.

CSE strongly influences student aspirations, motivation and accomplishment in higher education,^8^ and stems from four main sources: enactive mastery experiences, vicarious experiences, verbal persuasion, and physiological affective states.^9^, ^10^ *Enactive mastery experiences* are when a student reflects on the results of previous attempts at creativity, and are the most powerful source of self‐efficacy.^11^ *Vicarious experiences* are when students learn about their creative abilities indirectly, by comparing their creative outputs to those of peers.^9^ *Verbal persuasion* is another indirect way of increasing a student's CSE, when students are encouraged to view a creative task as achievable.^9^ *Physiological affective states* are psychosomatic phenomena and generate CSE if a student attributes their creativity to how they were ‘feeling’ at the time.^12^

### 1.2 Benefits of increasing student CSE

CSE affects the creative goals that students set for themselves (e.g., challenging vs. non‐threatening/facile), their performance expectations, and whether or not they put effort into their creative tasks. These beneficial behaviours are reason enough to promote the development of CSE in students, however, the importance of CSE is further highlighted when considering student stress, affect, and mood, with low CSE being associated with unhelpful cognitions (thoughts), affective responses (feelings), and behaviours.^8^

Stress and affect share a complex relationship, but often, students will experience negative affective responses (e.g., anger, contempt, anxiety) in academic contexts that they find chronically distressing, when (e.g., through a lack of CSE) they perceive themselves unable to meet the demands of the situation.^13^ While moderate stress levels can stimulate creativity, intense stress typically prevents creative thought.^14^ Hence, when students' creative abilities are assessed, educators should increase their CSE through structured ‘low evaluative’ activities and formative assessments that engender a sense of control.^15^ Ideally, teaching environments would be designed which regularly elicit learning experiences akin to ‘flow’ and ultimately, optimize creative performances when the stakes are highest, for instance during summative assessment.^16^, ^17^

‘Psychological capital’ (composed of hope, optimism, resilience, and importantly, self‐efficacy), correlates positively with creativity and the motivation to achieve, while correlating negatively with perceived stress.^18^ Similarly, positive affect – a consistently strong predictor of academic performance – has been shown to promote a proactive, approach motivation, which is influential in enhancing creativity and the use of creative cognition during study.^19^, ^20^ As might be expected, creative experiences and activities are also generally associated with health and wellbeing benefits for students, in addition to their academic benefits.^21^

### 1.3 Developing student CSE

The learning environment has a considerable impact on the development of students' CSE, and the CSE‐behaviour relationship.^22^ Behaviour is the interplay between personal factors (e.g., CSE) and the environment in which the behaviour is to be enacted – for example, the extent to which creativity has been role modelled by the lecturer, or the inclusion/omission of creativity/innovation in a module's learning outcomes.^10^, ^23^, ^24^ Many educators will intuitively engender supportive environments which are likely to stimulate the development of CSE, but there are relatively few published studies in the literature which specifically leverage the psychology of CSE in undergraduate science education.

This literature is mostly questionnaire‐based, determining the strength and direction of association between CSE and related constructs – such as science understanding, student‐teacher trust, transformational leadership, peers' creative abilities, intrinsic motivation, originality, creative self‐concept, and expressiveness – while also evidencing the ability of these constructs to predict CSE.^25^, ^26^, ^27^, ^28^, ^29^, ^30^, ^31^, ^32^, ^33^, ^34^, ^35^

CSE and related constructs can be directly targeted by intervention studies, yet very few intervention studies in higher education have been published. A few researchers have intervened and measured *creative output* as an outcome measure in a ‘snapshot’ fashion, but not targeted cognitive variables that are associated with *future* creativity, such as CSE. Of the few studies that intervened and assessed the resulting effects on CSE, none involved science undergraduates. Dewett and Gruys^36^ employed ‘unconventional’ teaching methods in an MBA course, in an attempt to improve creativity via increased comfort with risk and increased CSE. The intervention increased students' CSE, but interestingly, it did not significantly change how important students perceived creativity to be in the workplace, so it is unclear how sustainable such CSE increases are and what study/work practices they might prompt. Robbins and Kegley^37^ developed an online Creative Thinking Program founded on the principle of ‘psychological safety’ as a facilitator of creativity. They saw a statistically significant (but small) increase in CSE. Byrge and Tang^38^ administered an ‘embodied creativity training program’ and found that CSE can be enhanced through targeted intervention, while Mathisen and Bronnick^39^ investigated the power of creativity training on a variety of students, worker and teachers, and found that CSE increased and that the CSE increase persisted beyond the training course, compared to a control group.

### 1.4 Aims

No studies published to date have targeted the development of student CSE as part of the teaching of a STEM subject, despite CSE being linked to desirable student outcomes, such as the confidence to innovate, increased motivation to study, aspiration to succeed and increased academic attainment. The potential cognitive, motivational, affective, and behavioural benefits of increasing CSE led us to test the effectiveness of a theoretically‐derived teaching intervention designed to increase the CSE of undergraduate biochemistry students, and to investigate how CSE relates to biochemistry students' perceptions of their academic abilities and outcomes.

## 2 METHODS

### 2.1 Measures

A 19 item survey (File S1) was designed to gauge students' CSE and related aspects of their lives/studies. They were also asked to rate their attainment of ‘degree‐level’ learning outcomes and asked to predict their end‐of‐module mark. Survey items were derived from established theoretical propositions, for example, Bandura's^40^ guidance on the construction of self‐efficacy scales. Questions required responses in a variety of forms, including Likert scales, free text, and yes/no binary responses.

### 2.2 Procedure

#### 2.2.1 Study design and survey administration

Student participants were enrolled on Life Sciences BSc degrees. Three modules spanning the same semester were selected for inclusion: one, the ‘Intervention Module’ (BSc year 2, *n* = 20), contains an innovative creative element, while the other two ‘C*ontrol Modules*’ make no explicit efforts to develop student CSE (BSc years 1 and 3, combined *n* = 32). Ethical approval was obtained from the researchers' institution, informed consent was provided by students, and ethical principles ‐ as outlined by the American Psychological Association and British Psychological Society ‐ were adhered to throughout the study. Students were provided with the context for the study and a description of the data‐management plan for the project, and consented to complete early‐ and late‐semester questionnaires (weeks 1 and 11 respectively).

#### 2.2.2 CSE intervention

The creative exercise within the intervention module has been described previously.^41^ In brief, the module is practical‐based, with students undertaking a weekly series of biochemistry ‘wet lab’ experiments during the first half of the module, following staff‐provided experimental protocols. During the latter half of the module the students progress to design their own step‐by‐step experimental protocols (working in pairs), which they then implement in the laboratory, to determine how many atoms of iron there are in a molecule of hemoglobin (the ‘intervention’).^41^ Assessment of students was through their production of written reports for each experiment, including the experiment for which they developed their own protocols. Their protocols (i.e., the products of their creativity) were not assessed directly. The protocol‐design task was well‐scaffolded by constraining the available experimental materials, and with an engineered system of feedback and reflection. Students were set to work on the task in pairs for a week, after which they discussed their draft protocols with a member of staff. One week later they brought a revised draft to the laboratory, which was then prototyped by a pair of peers. Feedback from peers was reciprocal, interactive and happened in real‐time as the draft protocols were implemented. Finally, a week later a final version of the protocol was implemented by the protocol creators.^41^ The ‘intervention’ had been implemented for several iterations before this study and the both the module and the intervention assessment gave reproducible marks each year.

#### 2.2.3 Development of CSE

The intervention's scaffolding pedagogy, social elements, and in‐class feedback targeted the sources of CSE described previously (e.g., enactive mastery experiences, vicarious experiences and verbal persuasion).^9^, ^10^ Each student participant attended each feedback session, and to increase treatment fidelity care was taken to ensure each student received consistent and accurate feedback from staff, even during the peer‐prototyping sessions. The social interaction element of the intervention aimed to create an environment where students were inspired to act on their strengthening perception of CSE, and because social interactions promote academic achievement.^6^ The ‘low evaluative’ context and controllability engendered by the intervention are associated with lower stress and more effective creative performance than highly evaluative and uncontrollable creative environments.^15^

### 2.3 Data analysis

For the key study variable CSE, paired responses (beginning and end of the module) were obtained from 19 students on the intervention module, and from 31 students on the control modules, respectively. (For some between‐group analyses the full *n* of 20 and 32 were available.) Likert scale data was assumed to be a discretisation of an underlying continuous variable. The distribution characteristics of all data were evaluated using visual checks (histograms and normal *Q*–*Q* plots), skewness and kurtosis calculations, and statistical tests (Kolmogorov–Smirnov and Shapiro–Wilk). The Kolmogorov–Smirnov and Shapiro–Wilk tests tended to suggest non‐normal distribution, which was in conflict with observations derived from the other inspection methods. Hence, parametric (*t* tests) and non‐parametric (Mann–Whitney *U* or Wilcoxon *Z*) tests of difference, and parametric (Pearson's) and non‐parametric (Spearman's) tests of association (corrected for multiple tests), were conducted to provide a comparison of outcomes. The direction, strength, and significance of correlations were broadly similar for the parametric and non‐parametric tests, so the parametric tests are reported. Alpha was set at *p* < 0.05 for all one‐ and two‐tailed tests of significance.

## 3 RESULTS

### 3.1 Teaching interventions can give targeted increases in student CSE

Table 1 displays descriptive data for the study's main variables, while Table 2 summarises the early‐to‐late‐module *change* score comparisons between groups. The intervention group's CSE significantly increased from beginning to end of module (*t*_[18]_ = −2.970, *p* < 0.01; *x̄* = 3.00 to 3.53/5), whereas the control groups' CSE did not change significantly (*x̄* = 3.61 to 3.74/5). The difference between the two groups in CSE *change* from pre‐ to post‐ intervention (0.13 vs. 0.53) was also significant (*t*_[48]_ = 1.778, one‐tailed *p* = < 0.05).

**TABLE 1:** Average scores for the main study variables

	Control group			Intervention group		
Variable & item response scale range	Early	Late	Difference	Early	Late	Difference
Creative self‐efficacy (1–6)	3.61	3.74	+0.13	3.00	3.53	+0.53**
Creativity skills from past modules (1–4)	2.78	2.88	+0.10	2.05	2.90	+0.85***
Importance placed on creativity (1–6)	4.38	4.59	+0.21	4.45	4.65	+0.20
Strength of motivation to develop creative abilities (1–6)	4.38	4.31	−0.07	4.45	4.55	+0.10
Strength of motivation for university work (1–6)	4.09	3.61	−0.48	4.35	4.38	+0.03
Perceived ‘riskiness’ (−3 to +3)	1.00	0.88	−0.12	0.55	0.80	+0.25
Perceived impulsiveness (1–7)	4.72	4.50	−0.22	3.95	3.90	−0.05
*Creative STEMM abilities* (1–6):						
Novel protocols	3.84	4.06	+0.22	3.48	4.08	+0.60***
Novel data	3.50	3.91	+0.41*	3.75	4.40	+0.65**
Novel analyses	3.78	3.95	+0.17	3.50	4.15	+0.65*
Novel report	3.45	3.90	+0.45	4.00	4.63	+0.63**
Unfamiliar lab	3.75	3.69	−0.06	3.88	4.58	+0.70**
Expected module score (%)	61.3 (7.8)	63.3 (9.3)	+2%	60.0 (18.0)	63.6 (6.0)	+3.6%

* *p* < 0.05;

** *p* < 0.01;

*** *p* < 0.001.

**TABLE 2:** Average change in study variables from pre‐to‐post intervention

	Control group	Intervention group	
Variable & item response scale range	Early > late difference	Early > late difference	Significant between‐group difference?
Creative self‐efficacy (1–6)	+0.13	+0.53	*p* < 0.05
Creativity skills from past modules (1–4)	+0.09	+0.85	*p* < 0.01
Importance placed on creativity (1–6)	+0.21	+0.20	
Strength of motivation to develop creative abilities (1–6)	−0.07	+0.10	
Strength of motivation for university work (1–6)	−0.47	+0.03	
Perceived ‘riskiness’ (−3 to +3)	−0.12	+0.25	
Perceived impulsivity (1–7)	+0.02	−0.05	
*Creative STEMM abilities* (1–6):			
Novel protocols	+0.22	+0.60	*p* = 0.051
Novel data	+0.41	+0.65	
Novel analyses	+0.17	+0.65	
Novel report	+0.45	+0.63	
Unfamiliar lab	−0.06	+0.70	
Expected module score (%)	+2%	+3.6%	

When measured early and late in the semester, the importance participants placed on the need to be creative in their studies remained relatively constant in *both* groups (control: *x̄* = 4.38 and 4.59/6; intervention: *x̄* = 4.45 and 4.65/6), as did their motivation to develop their creative abilities (control: *x̄* = 4.38 and 4.31/6; intervention: *x̄* = 4.45 and 4.55/6). The early‐to‐late‐module *change* scores for these two variables did not differ between the two groups (*t*_(50)_ = −0.054, *p* > 0.05 and *t*_(50)_ = 0.426, *p* > 0.05, respectively). Hence, other between‐group comparisons (control vs. intervention) can be interpreted in light of the fact that the two groups did not differ in these underpinning variables, suggesting that the changes observed in CSE are a true reflection of the intervention's effectiveness and not an artefact associated with changes in the two variables above.

Interestingly, at pre‐intervention, only 10% of participants believed that they would know ‘where to start if they wanted to learn to be more creative’, but at post‐intervention this rose to 55%. Students were not explicitly trained in how to seek out creativity‐raising opportunities, so it seems that their attitude to developing their creativity had been improved by virtue of their involvement in the CSE intervention.

### 3.2 CSE increases coincide with enhanced perceptions of course creativity and attainment of learning outcomes

At the beginning and end of the module participants indicated the extent to which they believed they had developed effective creative skills on past modules during their degree. The intervention group's scores significantly increased on average (*t*_[19]_ = −4.344, *p* < 0.001; *x̄* = 2.05 to 2.90/4), whereas the control groups' did not (*x̄* = 2.78 to 2.88 / 4). This difference in *change* from pre‐ to post‐ between the two groups (0.09 vs. 0.85) was significant (*t*_(50)_ = 2.729, *p* < 0.01) and suggests that an increase in contemporary CSE can retrospectively increase students' perceptions of the historical development of their creativity.

Students also rated themselves on the ability to meet five ‘degree‐level’ learning outcomes related to creativity (‘How would you rate your own ability to … produce novel protocols for use in a laboratory’, ‘generate novel and useful experimental data’, ‘analyse and interpret novel data in a useful manner’, ‘produce a report of novel data and its interpretation, for use by other scientists’, and ‘step into an unfamiliar laboratory and work productively’). Between the two surveys, participants in the control group gained a significantly improved perception of their ability to generate novel and useful experimental data (*x̄* = 3.50 to 3.91/6; *t*_[31]_ = −2.204, two‐tailed *p* < 0.05), but no other perceived abilities changed significantly. In contrast, and mirroring the observed increases in their CSE, the intervention group gained a significantly improved perception of *all* five abilities (increases ranging from 0.60 to 0.70/6; all one‐tailed *p* values = <0.01).

### 3.3 Contributory factors to academic CSE and a disparity with extra‐curricular CSE

Participants were asked on what experiences and thoughts their academic creative ability self‐ratings were based. When asked early in the module, students cited previous successful experiences as the primary positive influence, while negative perceptions were based on previously unsuccessful experiences (File S2).

Participants were also asked whether their perceived creativity in their studies differed from other life contexts, and if so, why. Prior to the intervention 55% of students from the intervention module indicated that their academic CSE was relatively lacking. Example rationales are provided in File S2. However, at post‐intervention, 75% said they would rate their CSE similarly within university and beyond, indicating that for the intervention group, improved academic CSE self‐rating had begun to converge with their CSE from contexts in which they were more comfortable/confident (File S2).

### 3.4 Positive relationships between CSE, student‐predicted grades and academic attainment

The intervention took place in a year two module of a 3 year degree program, therefore participants' year one average scores were available to the researchers, as were their scores from the module itself upon its completion. At pre‐ and post‐intervention, participants were asked to estimate the score they expected to achieve on the module (and what informed their estimations – see File S2). A series of correlation tests were performed to determine the strength and direction of relationship between actual scores, expected scores, and CSE (Table 3).

**TABLE 3:** Relationships between CSE, performance expectations, and actual performance (*n* = 14)

	*Performance indicators and CSE variables*					
*r* values	Year one performance (*x̄* = 65%, SD = 5)	Pre‐intervention expected module score (*x̄* = 60%, SD = 18)	Post‐intervention expected module score (*x̄* = 64%, SD = 6)	Intervention module actual performance (*x̄* = 65%, SD = 7)^a^	Pre‐intervention CSE (*x̄* = 3.07, SD = 0.73)	Post‐intervention CSE (*x̄* = 3.64, SD = 0.50)
Year one performance (*x̄* = 65%, SD = 5)						
Pre‐intervention expected module score (*x̄* = 60%, SD = 18)	−0.427 (one‐tailed *p* > 0.05)					
Post‐intervention expected module score (*x̄* = 64%, SD = 6)	0.766 (one‐tailed *p* < 0.001)	−0.148 (one‐tailed *p* > 0.05)				
Intervention module actual performance (*x̄* = 65%, SD = 7)*	0.630 (one‐tailed *p* < 0.01)	−0.263 (one‐tailed *p* > 0.05)	0.524 (one‐tailed *p* < 0.05)			
Pre‐intervention CSE (*x̄* = 3.07, SD = 0.73)	0.550 (two‐tailed *p* < 0.05)	0.174 (two‐tailed *p* > 0.05)	0.550 (two‐tailed *p* < 0.05)	0.314 (two‐tailed *p* > 0.05)		
Post‐intervention CSE (*x̄* = 3.64, SD = 0.50)	Not needed	Not needed	0.587 (one‐tailed *p* < 0.05)	0.376 (one‐tailed *p* = 0.093)	0.076 (one‐tailed *p* = >0.05)	

^a^ For analyses related to the intervention group's performance data, five participants were removed because they did not achieve their full potential due to non‐submission of one or more coursework components; *n* = 14.

Grey shades indicate comparisons which would be self‐self.

At pre‐intervention, students generally predicted that they would achieve a statistically similar module mark to that which they achieved overall in their first year (60% vs. 65%, respectively). However, despite the similar means, the correlation between the two variables was moderately strong and negative (*r* = −0.427, *p* < 0.05) suggesting a disconnection between past experiences and future expectations. Nevertheless, at post‐intervention the students' predicted marks had risen slightly (*x̄* = 63.6%, SD = 6.0), aligning more closely to their actual module score (*x̄* = 64.7%, SD = 7.0).

In terms of CSE, the relationship between pre‐intervention and post‐intervention CSE was almost non‐existent (*r* = 0.076, one‐tailed *p* > 0.05), suggesting independence between the two variables and further strengthening the study's justification (Table 3). The relationship between post‐intervention CSE and actual module score was stronger but did not quite achieve statistical significance (*r* = 0.376, *p* = 0.093). Nevertheless, CSE did demonstrate a positive relationship with module performance, suggesting its status as a contributing factor.

### 3.5 CSE correlates with increased motivation to study and may also impact on risk‐taking behaviour

Students experienced some interesting and potentially beneficial changes associated with their participation in the CSE intervention. Control group participants' strength of motivation for university work substantially decreased from beginning to end of the semester (*x̄* = 4.09 to 3.61/6; *t*_[31]_ = 1.789, two‐tailed *p* = 0.083), whereas the intervention groups' improved slightly (*x̄* = 4.35 to 4.38/6; *t*_[19]_ = −0.101, *p* > 0.05). Late in the module, the intervention group's increased CSE shared a statistically significant, strong, positive relationship (*r* = 0.690, two‐tailed *p* < 0.001) with their (slightly increased) strength of motivation for university work (*x̄* = 4.38/6). For the control group, late in the semester, there was no relationship between their CSE and their motivation for university work (*r* = −0.103, *p* = 0.581). It seems plausible that the intervention, which required on‐going completion of a series of tasks which clearly built on one another, stimulated students' prolonged engagement (and motivation) as well as developing their CSE.

At the beginning and end of the module participants were also asked how ‘risky’ (i.e., ‘prone to take a course of action when the outcome is far from certain’) and impulsive (i.e., ‘prone to act before giving the consequences full consideration’) they would say they were in everyday life. Statistically, participants in the control group maintained their perceived ‘riskiness’ (*x̄* = 1.00 and 0.88/−3 to +3) and impulsiveness (*x̄* = 4.72 and 4.50 / 7), as did the intervention group (*x̄* = 0.55 and 0.80/−3 to +3; *x̄* = 3.95 and 3.90/7, respectively; all two‐tailed *p* values >0.05). However, decreases in the average raw scores for riskiness and impulsiveness can be seen for the control group but less so for the intervention group. The intervention sought to encourage risk‐taking and creative exploration through careful scaffolding and peer‐to‐peer support, and this observation is therefore noteworthy, as students often see creativity and risk‐taking as synonymous, preferring to play‐it‐safe rather than be creative/take a risk.^42^

## 4 DISCUSSION

### 4.1 An intervention targeted to increase student CSE

The majority of life science undergraduates we surveyed (55%) had lower CSE in the context of their studies than in extra‐curricular contexts. However, an intervention which targeted the development of CSE increased students' academic CSE, bringing it more in line with their extra‐curricular CSE. Intervention module students and control module students exhibited no significant differences prior to the intervention, and both sets of students were ‘primed’ equally. The increase in CSE of the intervention module students was not due to an increased perception of the importance of creativity, or the motivation to develop creativity, as these remained stable pre‐ and post‐intervention (Table 1).

The intervention was designed to draw on factors known to promote CSE, such as a classroom environment that was conducive to non‐threatening peer‐to‐peer collaboration. By scaffolding the students' learning activities, the module provided low‐stakes ‘creative space’ for enactive mastery experiences to occur. This approach was reinforced through a system of reflection and feedback, which is a valuable contributor to students' introspection and metacognition.^9^, ^10^ Personal reflections on learning and performance, integrated with feedback from an expert, should result in lasting increases in CSE as an indirect form of enactive mastery experience.^39^ It would therefore be interesting to investigate whether the ability to self‐reflect is a mediator of the efficacy of this intervention.

Peer‐to‐peer support was also integral to the intervention, with classmates providing a source of CSE through verbal persuasion. Classmates worked together to critique their own and their peers' creative process and output, which would be expected to facilitate positive mood states and enactive mastery through vicarious experience and external perspective‐taking.

### 4.2 Benefits associated with increases in CSE

The increased CSE resulting from the intervention was associated with other beneficial changes in the students' survey responses. Compared to students on the control modules, post‐intervention the intervention group exhibited increased motivation and risk‐taking, with increased confidence in their ability to achieve learning outcomes and to enter the workplace (Table 1).

Increases in various types of academic self‐efficacy (e.g. reading self‐efficacy) have been shown to be associated with improvements in that type of academic performance.^43^, ^44^ Unsurprisingly, CSE is a contributing factor to creative performance, albeit the strength of that contribution can increase, remain stable, or decline over time.^12^, ^45^ It would be interesting to test whether increases in CSE as a result of this intervention are also associated with, or cause, an increase in creativity. However, as self‐efficacy and performance are reciprocally deterministic, a ‘chicken‐and‐egg conundrum’ exists, making attempts to establish causality difficult.^46^

Increased CSE was associated with increased motivation for study at the end of the semester (Table 1). This is important because completion of assignments, class attendance, hours spent studying, and final grade, are all associated with motivation.^47^, ^48^, ^49^ Motivation to study is time‐dependent, for instance, how students compare themselves to peers is a motivational factor that evolves with time.^50^ All‐too‐often, the strength and/or quality of student motivation is diminished from the start to the end of a semester,^50^, ^51^, ^52^, ^53^ but this was not observed in the intervention group. Potentially, the tempo of the intervention, with practical tasks being completed on an ongoing basis clearly building on one another, stimulated the students' prolonged engagement.

### 4.3 Areas for further consideration

Increases in CSE can be lasting,^39^ and it would be interesting to undertake longitudinal studies to assess the frequency with which CSE‐promoting interventions should be embedded in the curriculum. Will students with increased CSE act on their increased sense of familiarity with the creative side of their subject to further develop their own creativity?

It would also be interesting to explore the relationship between CSE and perceptions of risk‐taking in students. Risk‐taking is domain specific^54^ and future research should investigate how risk and impulsiveness as specific to academic pursuits, relate to student perceptions of creativity and CSE. Scaffolded opportunities for creative and innovative exploration may attenuate students' belief in the ‘riskiness’ of creative behaviours – potentially resulting in an altered approach, from avoidance of risk to pursuit of opportunity.^55^

The nature of the intervention we deployed in this study meant that our sample size *n* was necessarily small, although *n* was sufficient to demonstrate some significant differences between intervention and control groups and between early/late or pre‐/post‐intervention. The conclusions drawn here are therefore somewhat tentative, and it would be useful to target further larger modules with bespoke CSE‐targeted interventions to establish the generality of our observations, which would likely extend beyond biochemistry to other STEM subjects and/or school education settings.

It is concerning that there was a negative correlation between students' prior marks and the pre‐intervention prediction of their marks in the intervention module (Table 3). It could be that students were threatened by the creativity element of the module, and this manifested in a pessimistic score prediction, potentially also as a protective self‐presentational strategy.^56^, ^57^

Nevertheless, an increased correlation between post‐intervention predictions and marks obtained for the intervention module (Table 3), suggest the intervention may help students link learning activities to learning outcomes and performance. Students performed better than they had expected to at the start of the semester, also supporting the intervention's effectiveness of ‘coaching up’ students' creative abilities. In our experience ‘creativity‐related’ terms are usually missing from marking criteria and learning outcomes, making it difficult for students to reconcile creativity with scoring points for performance.

It is also noteworthy that many students' reported that their pessimistic predictions of module marks were due to previous negative experiences (Supplemental File S2). This reinforces the importance of making creative exercises low stakes and non‐threatening, otherwise creative teaching exercises could be counterproductive, reducing students' CSE.^58^

### 4.4 Implications for practice

CSE is an important component of the student experience that can be actively enhanced by pedagogic strategies, although in higher education there is often little opportunity for the required ‘risk‐taking, collaborative exploration and autonomy’,^59^ and creative exercises also have to compete for time with the taught curriculum.^60^ Pedagogic interventions aside, leader CSE has an indirect effect on the creativity (if not the CSE) of followers,^61^, ^62^ and academics can role‐model creativity, encouraging engagement of their students with the creative process.

The teaching intervention we deployed did not require teaching staff to have strong CSE of their own. Instead, it included (i) learning tasks designed to increase students' CSE as well as a mode of assessment that inspired creative efforts, and (ii) creation of a learning environment that provided enabling resources, opportunity structures, and was intended to reciprocally benefit students' CSE. These are relatively simple teaching interventions that are grounded in strong psychological theory, that target the four key sources of CSE (enactive mastery experiences, vicarious experiences, verbal persuasion, and physiological affective states).^10^, ^23^, ^24^ Such interventions can be relatively simple and straightforward to implement. For example, by providing ‘practice sessions’ where students can access the laboratory to informally experiment with techniques and equipment, by facilitating discussions between students to compare or aggregate protocols/data, or by breaking up challenging tasks into smaller sub‐tasks which can then be brought together when the individual sub‐tasks have been mastered.

Potentially, the composition of the peer group element of the intervention could be manipulated to increase beneficial outcomes. Should students be given autonomy to choose who to team up, or should they be placed into groups that challenge their zone of proximal development and role model effective study habits^63^? Composition of the peer groups also has implications for stress levels, social anxiety, risk‐taking, and engagement behaviours,^64^ and so warrants further investigation if CSE‐raising interventions are to maximise their effectiveness.

Many excellent resources are available to help educators promote creativity in their teaching.^65^, ^66^ Unfortunately, teaching practices such as modelling creativity, allowing time for creative thinking, allowing mistakes, and rewarding creative outputs, for example, are often forgotten by students as their deadlines loom and they revert to marks‐acquisition mode to the exclusion of skill development.^67^ Potentially, inclusion of ‘innovation’ and ‘creativity’ terms throughout assessment marking criteria and module learning outcomes, would help students to see the impact of creativity on their marks, and the value that teaching staff and employers place on creativity and innovation. Ideally, teachers would also design their modes of assessment to explicitly include some opportunity for creative thought and expression,^68^ harnessing the benefits of CSE‐associated study behaviours.^10^, ^69^, ^70^, ^71^

## Supporting information


**File S1** A survey, deployed pre‐intervention and post‐intervention for the intervention module, and early and late in the semester for control modules.

**File S2** Illustrative examples of student responses to open‐ended survey questions.
