# Supplementary material for: Increasing creative self‐efficacy: Developing the confidence of biochemistry undergraduates to innovate
Source: Biochem Mol Biol Educ. 2022 Apr 23;50(3):296–306. doi: 10.1002/bmb.21628 (PMC9321695; doi:10.1002/bmb.21628)
Supplement: Supplementary file 1 — File S1 A survey, deployed pre‐intervention and post‐intervention for the intervention module, and early and late in the semester for control modules. [file BMB-50-296-s002.docx]

**Creativity and Creative Self-Belief**

We would like to invite you to participate in a research programme looking into students’ perspectives on various aspects of their learning. The results from this research will help staff to improve their teaching. This particular survey is concerned with (1) ‘creativity,’ commonly defined as the ability to create novel and useful ideas &/or products, and (2) ‘creative self-belief,’ which refers to one’s perceived ability to create novel and useful ideas &/or products. Your response to this survey will remain absolutely confidential, and you will not be named or identifiable in any outputs of the research. We will be asking a few more questions at a later date and so we require your student number so that we can keep your responses together in the dataset. Please be completely honest, and the best way to ensure this is to respond with your immediate feeling.

**Student Number:**

1. In the context of your degree scheme how would you rate your own ability to generate *novel* and *useful* ideas/products?

1 2 3 4 5

Very Poor Average Good Very

poor good

- 1. Does this rating differ from how you would rate your ability to generate *novel* and *useful* ideas/products in other life contexts? Yes / No
  2. If Yes, could you briefly explain below how and why it differs?

1. In your life before Aber Uni &/or in your life outside your degree scheme have you been (or are you) involved in activities that require creativity? (i.e., the generation of novel and useful ideas and products) Yes / No
   1. If Yes, could you briefly describe the activities?
2. Please use this scale to indicate whether you have developed effective creative skills on past modules on your degree?

1 2 3 4

Not Somewhat I’m pretty Yes, definitely

at all happy

1. Please use this scale to indicate the degree of importance you place on the need to be creative in your academic practices?

1 2 3 4 5 6

Of no Extremely

importance important

1. How strong is your motivation to develop your creative abilities?

1 2 3 4 5 6

Extremely Extremely

weak strong

1. Would you know where to start if you wanted to learn to be *more* creative? Yes / No
2. Please describe in one sentence what motivates you to study, learn, and strive to achieve your academic goals:
3. How strong is your motivation for uni work right now?

1 2 3 4 5 6

Extremely Extremely

weak strong

1. In general, how ‘risky’ would you say you are? (i.e., prone to take a course of action when the outcome is far from certain)

-3 -2 -1 0 1 2 3

Risk- Risk- Risk-

Averse Neutral Seeking

1. In general, how impulsive would you say you are? (i.e., prone to act before giving the consequences full consideration)

1 2 3 4 5 6 7

Not at all Somewhat Extremely impulsive impulsive impulsive

**And finally,**

On a scale of 1-6, where:

1 2 3 4 5 6

Very Poor Below Above Good Very

poor average average good

How would you rate your own ability to:

1. Produce novel protocols for use in a laboratory?
2. Generate novel and useful experimental data?
3. Analyse and interpret novel data in a useful manner?
4. Produce a report of novel data and its interpretation, for use by other scientists?
5. Step into an unfamiliar laboratory and work productively?

Finally,

1. What mark do you expect to get for this module?
2. Please very briefly describe on what experiences or thoughts the above self-ratings are based:
