# Supplementary material for: Increasing creative self‐efficacy: Developing the confidence of biochemistry undergraduates to innovate
Source: Biochem Mol Biol Educ. 2022 Apr 23;50(3):296–306. doi: 10.1002/bmb.21628 (PMC9321695; doi:10.1002/bmb.21628)
Supplement: Supplementary file 2 — File S2 Illustrative examples of student responses to open‐ended survey questions. [file BMB-50-296-s001.docx]

**Illustrative Qualitative Responses**

**Contributory factors to academic CSE and a disparity with extra-curricular CSE**

Participants were asked on what experiences and thoughts their academic creative ability self-ratings were based. When asked early in the module, students cited previous successful experiences as the primary positive influence (“from ... having designed a successful protocol in [a previous module],” “From writing protocols in previous modules”). Similarly, negative influences included previous unsuccessful experiences (“I find lab work nerve-wracking and a lot more difficult to learn from. It takes me significantly longer to understand lab protocols and results than other ways of investigation,” “I'd like to think I'm good at coming up with experiments, however my protocols are flawed” “The last time I wrote a protocol it failed and was awful. Experiments always go wrong”).

Participants were also asked whether their perceived creativity in their studies differed from other life contexts, and if so, why. Prior to the intervention 55% of students from the intervention module indicated that their academic CSE was relatively lacking. Rationales included “Different ways of thinking about ideas in scientific writing than everyday contexts,” “I find it difficult to create ideas/products relating to subjects where I still have a lot to learn,” and “I have more chances/freedom to express my ideas in other areas.” However, at post-intervention, 75% said they would rate their CSE similarly within university and beyond, indicating that for the intervention group, improved academic CSE self-rating had begun to converge with their CSE from contexts in which they were more comfortable/confident (including e.g., baking, art, fundraising, amateur dramatics, entrepreneurship, carpentry, and designing activities for clubs and societies).

**Positive relationships between CSE, student-predicted grades and academic attainment**

At pre- and post-intervention, participants were asked to estimate the score they expected to achieve on the module and what informed their estimations.

When asked post-intervention on what their performance predictions were based, students’ qualitative responses attested to the reinforcing power of feedback (“I learned a lot from my mistakes”), meaningful effort and associated positive psychosomatic states (“I feel pretty good about it!”), and the inherent benefits of experience (“realisation of the importance of creativity in a scientific context.”).

**CSE correlates with increased motivation to study and may also impact on risk-taking behaviour**

Participants were asked to describe, in one sentence, what motivated them to study, learn, and strive to achieve their academic goals. Major themes were the desire to get a get a job after graduation and the drive to excel (“to become a more academic individual and to achieve a better job”, “in order to gain the experience I need for a job to potentially use the skills I have learned during my degree”).
